# Progress in Manipulating Dynamic Surface Reconstruction via Anion Modulation for Electrocatalytic Water Oxidation

**DOI:** 10.1002/advs.202304071

**Published:** 2023-08-08

**Authors:** Zexing He, Muhammad Ajmal, Minghui Zhang, Xiaokang Liu, Zhen‐Feng Huang, Chengxiang Shi, Ruijie Gao, Lun Pan, Xiangwen Zhang, Ji‐Jun Zou

**Affiliations:** ^1^ Key Laboratory for Green Chemical Technology of the Ministry of Education, Institute of Molecular Plus, School of Chemical Engineering and Technology Tianjin University Tianjin 300072 China; ^2^ Collaborative Innovative Center of Chemical Science and Engineering Tianjin University Tianjin 300072 China; ^3^ Zhejiang Institute of Tianjin University Tianjin University Ningbo Zhejiang 315201 China

**Keywords:** anion modulation, catalyst design, electrocatalytic water oxidation, reaction mechanism, surface reconstruction

## Abstract

The development of efficient and economical electrocatalysts for oxygen evolution reaction (OER) is of paramount importance for the sustainable production of renewable fuels and energy storage systems; however, the sluggish OER kinetics involving multistep four proton‐coupled electron transfer hampers progress in these systems. Fortunately, surface reconstruction offers promising potential to improve OER catalyst design. Anion modulation plays a crucial role in controlling the extent of surface reconstruction and positively persuading the reconstructed species' performances. This review starts by providing a general explanation of how various types of anions can trigger dynamic surface reconstruction and create different combinations with pre‐catalysts. Next, the influences of anion modulation on manipulating the surface dynamic reconstruction process are discussed based on the in situ advanced characterization techniques. Furthermore, various effects of survived anionic groups in reconstructed species on water oxidation activity are further discussed. Finally, the challenges and prospects for the future development directions of anion modulation for redirecting dynamic surface reconstruction to construct highly efficient and practical catalysts for water oxidation are proposed.

## Graphical abstract

Anion modulation represents a potential direction for controllable extent of surface reconstruction and enhancement of reconstructed species' performances. This review summarizes various origins of anions adsorbed on reconstructed catalysts and influences of anion modulation on manipulating the surface dynamic reconstruction process as well as reconstruction species. Furthermore, invigorating development orientation of anion modulation for manipulating surface reconstruction is provided.

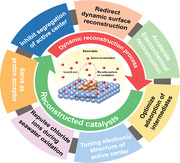

## 1 Introduction

Human society is confronted with a global energy crisis concomitant with the gradual depletion of finite fossil fuels.^[^ ^1^ ^]^ The renewable energy source (e.g., solar, wind, and geothermal) is swiftly emerging as a key technology to mitigate the impact of this crisis and decarbonizing parts of the energy system.^[^ ^2^ ^]^ Hydrogen energy with high gravimetric energy density holds immense potential as a renewable energy carrier and a viable alternative to conventional fossil fuels, which can accelerate dcarbonization process and transformation of energy consumption structure.^[^ ^3^ ^]^ In the context of decreasing cost of electricity derived from renewable energy, electrochemical water splitting is emerging as an exceedingly captivating pathway for large‐scale green hydrogen production.^[^ ^4^ ^]^

**Figure 1:**
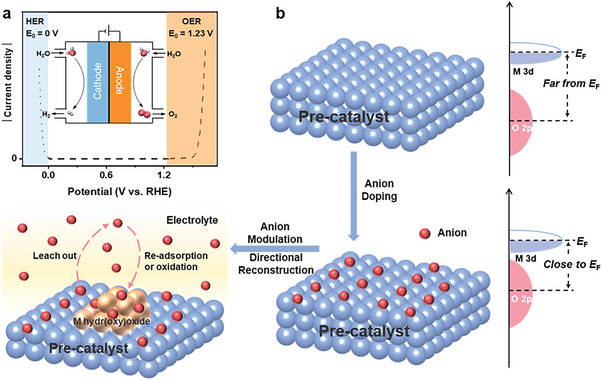
a) Illustration of electrochemical water splitting reactions. b) Schematic diagram of manipulating dynamic reconstruction process of pre‐catalyst with anion modulation.

Water splitting is made up of two half‐reactions including hydrogen evolution reaction (HER) and oxygen evolution reaction (OER). Different from HER, OER commonly imposes constraints on the overall efficiency owing to its sluggish reaction kinetics, involving complex multistep proton‐coupled electron transfer and spin constriction (**Figure** **1**a).^[^ ^5^ ^]^ To address this constraint, there has been extensive enthusiasm in the realm of designing and fabricating electrocatalysts endowed with exceptional catalytic activity and enduring long‐term durability for OER.^[^ ^6^ ^]^ Various materials have been explored, including metal oxides,^[^ ^7^ ^]^ layered double hydroxides (LDHs),^[^ ^8^ ^]^ organic frameworks (MOF),^[^ ^9^ ^]^ oxyhydroxide,^[^ ^10^ ^]^ sulfides,^[^ ^11^ ^]^ and alloys.^[^ ^12^ ^]^ Moreover, with the help of in situ/operando characterization, such in situ Raman spectra, in situ Fourier transform infrared (FT‐IR) spectra and differential electrochemical mass spectrometry (DEMS) etc, recent studies have demonstrated that most of these compounds, called as “pre‐catalysts”, are not the real active sites. Instead, they inevitably undergo different degrees of surface reconstruction into metal hydroxides (M(OH)_x_) and oxyhydroxides (MO_x_H_y_), which are regarded as actual active species during the OER process.^[^ ^13^ ^]^ Consequently, significant endeavors have been dedicated to the regulation and promotion of surface reconstruction.^[^ ^14^ ^]^ However, the quest for achieving precise control over surface reconstruction persists as a formidable challenge, because the complexity lies in the fact that surface reconstruction can be influenced by various factors, including internal factors (the composition and structure of pre‐catalyst) and external factors (e.g., pH value of electrolyte, applied potential, and electrolyte ions, etc).^[^ ^15^ ^]^

The strategies of regulating surface reconstruction including anion modulation, cation leaching and defect engineering, etc. It's difficult for most of them to precisely tune the surface reconstruction. Recently, extensive investigations have proven that anion modulation can effectively manipulate the degree of dynamic surface reconstruction for pre‐catalysts and construct a highly efficient electrocatalytic environment for reconstructed species to improve the OER reaction kinetics (Figure 1b).^[^ ^16^ ^]^ The incorporation of anionic groups can induce significant alterations in the microscopic morphology, electronic structure, adsorption and desorption behaviors of reaction intermediates and even reaction mechanism of reconstructed catalysts.^[^ ^17^ ^]^ For example, Zhang and co‐workers reported an intriguing phenomenon of self‐reconstruction in surface sulfides under OER conditions. The sulphides transformed into oxysulphides with a multi‐anionic structure, which have been validated as the active sites for the reaction.^[^ ^18^ ^]^ Furthermore, they developed an electrochemical assisted approach to generate the anionic‐regulated NiFe hydroxysulfides in a Li‐S cell, unveiling exceptional electrocatalytic activity.^[^ ^19^ ^]^ Surprisingly, due to the presence of chloride ions LiCoO_2‐x_Cl_x_ reconstructs its surface into unique amorphous Cl‐doped cobalt(oxy)hydroxide phase, which is different from the original phase transformation pathway of LiCoO_2.0_ during OER process.^[^ ^20^ ^]^ Consequently, the catalyst reconstructed in such specific manner exhibits superior electrocatalytic activity and durability in comparison to the un‐doped counterpart.

Although some excellent reviews on the topic of electrochemical reconstruction^[^ ^21^ ^]^ or anion‐regulated metal compounds^[^ ^22^ ^]^ for OER have been published in recent years, there is no specific review that comprehensively covers the correlations between anion modulation, dynamic surface reconstruction process and the OER performance. Therefore, this review aims to provide valuable insights into developing highly efficient and stable catalysts via anion modulation from the perspective of “clarifying reconstruction mechanism”‐“analyzing reconstruction features”‐“constructing directional reconstruction”, which provides an important support for the rational design and controllable preparation of oxygen evolution catalysts with industrial application prospects. First, we summarize origins of anions adsorbed on the reconstructed catalysts to provide audience with the guideline for the rational design of anion‐modified electrocatalysts. Meanwhile, different combination patterns between anions and pre‐catalysts are enumerated, including electrostatic interaction, hydrogen bonding, chemisorption interaction and covalent interaction. Afterward, we highlight the influences of anion modulation in manipulating surface dynamic reconstruction process, which can be monitored by the in situ advanced spectroscopic characterization techniques. Furthermore, various effects of anion modulation on reconstructed electrocatalysts for the enhanced OER activity are discussed in detail including optimizing the adsorption of oxygenated intermediates, tuning electronic structure of active center, repelling chloride ions during seawater oxidation, serving as proton acceptor and inhibiting the segregation of active center. Finally, the remaining challenges and perspectives of anion modulation in manipulating dynamic surface reconstruction are highlighted and we look forward that this review can provide critical insights for the development of more effective reconstructed electrocatalysts through engineering of anion modulation.

## 2 Origin of Anions Adsorbed on the Reconstructed Catalysts

To synthesize anion‐modified reconstruction species for boosting OER activity, various strategies have been widely employed, such as anions intercalation into the interlayer spaces of LDH catalysts,^[^ ^23^ ^]^ in situ phase transformation of MOFs in alkaline solutions^[^ ^24^ ^]^ and in situ electrochemical oxidation of the transition metal chalcogenides^[^ ^25^ ^]^ or phosphides,^[^ ^26^ ^]^ etc. Among these strategies, the origin of anions can be broadly classified into two groups, which will be discussed in the following sections. The first group consists of surface‐adsorbed anions originating from the chemical synthesis of pre‐catalysts, which remain unoxidized or incomplete oxidation under OER conditions. The other group involves anions that can be subsequently adsorbed onto the reconstructed catalysts from anodic electrooxidation or electrolyte.

### 2.1 Anions from the Chemical Synthesis of Pre‐Catalysts

#### 2.1.1 The Introduction of Anions during Chemical Synthesis Process

**Figure 2:**
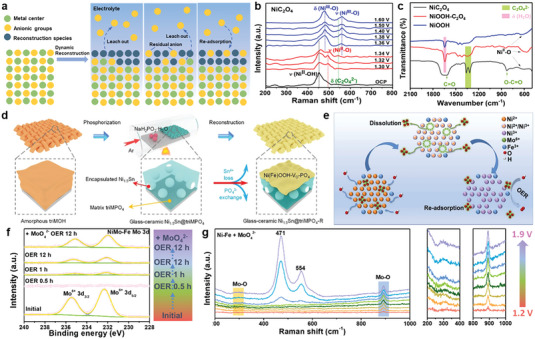
a) Scheme of different anion modulation modes during dynamic reconstruction process. b) In‐situ Raman spectra of NiC_2_O_4_. c) FT‐IR spectra of NiC_2_O_4_, NiOOH‐C_2_O_4_ and NiOOH. Reproduced with permission.^[^ ^31^ ^]^ Copyright 2023, Elsevier. d) Illustration for the synthesis of glass‐ceramic Ni_1.5_Sn@triMPO_4_ and its self‐reconstruction into (Ni(Fe)OOH‐Vo‐PO_4_). Reproduced with permission.^[^ ^33^ ^]^ Copyright 2021, Wiley‐VCH. e) Scheme of the dynamic dissolution and re‐adsorption process of MoO_4_^2−^ in the electrolyte. f) The Mo 3d XPS of NiMo‐Fe after different time of OER in KOH and in MoO_4_^2−^ contained KOH. g) In situ Raman spectra of Ni‐Fe in MoO_4_^2−^ contained KOH. Reproduced with permission.^[^ ^28^ ^]^ Copyright 2022, Elsevier.

Anions can be induced into lattice of pre‐catalysts via chemical coordination during chemical synthesis process, like solvothermal reaction,^[^ ^27^ ^]^ electrochemical procedure,^[^ ^28^ ^]^ and ion‐exchange method^[^ ^29^ ^]^ and so forth. As shown in **Figure** **2**a, several types of anions from pre‐catalysts can promote the formation of reconstruction species but continuously leach out and won't participate in the OER process.^[^ ^29^, ^30^ ^]^ Nonetheless, some anions can persistently associate with reconstructed catalysts, aiding the active sites in promoting OER process. During surface structural reconstruction, the pre‐catalysts can adopt a distinctive “core‐shell” structure, wherein anions‐functionalized bulk phase acts as core while reconstructed species act as shell.

In addition, during the synthesis process, certain anions can tightly adhere to the transition metal, consequently impacting both the reconstruction process and OER activity. For example, Zou et al synthesized NiC_2_O_4_ by precipitation reaction to generate C_2_O_4_‐modified NiOOH (NiOOH‐C_2_O_4_) through its directional reconstruction.^[^ ^31^ ^]^ The in situ Raman spectra and FT‐IR spectra demonstrate that C_2_O_4_^2−^ is not entirely substituted by OH^−^ during the reconstruction process of NiC_2_O_4_ into γ‐NiOOH (Figure 2b,c). And theoretical calculation reveals that surface‐adsorbed C_2_O_4_^2−^ on NiOOH acts as a critical and tunable proton acceptor, which plays a decisive role in reducing the deprotonation energy barrier. Song and co‐workers have also unveiled the anionic self‐optimization behavior of S‐Ni(OH)_2_, where the incorporation of Sulphur (S) with in the material is attributed to the presence of Sulfate ions (SO_4_^2−^) during synthesis process.^[^ ^32^ ^]^

Moreover, some anions in the lattice of pre‐catalysts tend to leach out during the reconstruction process. However, the residual anions with low content on the surface, which are often neglected, can contribute to excellent electrocatalytic activity. Therefore, the significance of surface‐adsorbed anions during reconstruction process should be further clarified. Wang and co‐workers synthesized defect‐rich amorphous Ni(Fe)OOH layer with intrinsic oxygen‐vacancies and residual phosphate anions (PO_4_^3−^) through the surface construction of Ni_1.5_Sn@triMPO_4_ under OER conditions (Figure 2d).^[^ ^33^ ^]^ The P 2p signal in XPS data confirms PO_4_^3‐^ ions are partially hydroxylized and the remaining sustains as residual PO_4_^3−^. The residual PO_4_^3−^ modulates the electron state of Ni site for improved OER activity. Another pioneering work by Li's group prepared the NiFe (oxy) hydroxides with different amounts of S residues by controlling the oxidation degree of the sulfides precursor.^[^ ^34^ ^]^ The elemental composition of all studied samples measured by energy dispersive X‐ray spectroscopy (EDX) reveals that the atomic S contents decrease by nearly two orders of magnitude after NiFe sulfide being converted to NiFe (oxy)hydroxide. However, the residual S is still proved to play a vital role in the OER activity of NiFe (oxy) hydroxides.

It's worth noting that several special anions can experience dynamic dissolution and re‐adsorption on the surface of reconstructed species. For example, Pan and co‐workers reported the synthesis of amorphous Fe‐incorporated NiMo oxyhydroxide via electrochemical procedure.^[^ ^28^ ^]^ The in situ Raman spectra and XPS analyses uncover the escape of the almost whole MoO_4_^2−^ into the electrolyte and the dynamic re‐adsorption behavior of the dissolved MoO_4_^2−^ (Figure 2e–g). The dissolution of MoO_4_^2−^ intensifies the reconstruction of pre‐catalyst, leading to the formation of active species, meanwhile re‐adsorbed MoO_4_^2−^ can further boost OER activity by enhancing the adsorption of the OOH* intermediate.

#### 2.1.2 Chemical Ligand‐Induced Conversion of LDHs

LDHs, composed of earth abundant elements, have been identified to be one of the most promising water oxidation pre‐catalysts due to the favorable electronic structure of cationic metals.^[^ ^35^ ^]^ The catalytic efficiency of LDH materials can also be enhanced by phase transformation behavior during OER process at high anodic potentials.^[^ ^36^ ^]^ However, LDH materials encounter challenges due to slow and incomplete reconstructions, which make the process of achieving the stable and best OER activity arduous.^[^ ^37^ ^]^ Ligand‐induced conversion strategy is used to regulate the degree of electrochemical reconstruction and boost OER activity of LDH materials by anions modulation, including anion exchange,^[^ ^38^ ^]^ in situ intercalation^[^ ^39^ ^]^ and phase transformation of MOFs,^[^ ^40^ ^]^ etc. The most thermodynamically stable interlayer anion of LDHs is carbonate (CO_3_^2−^) and in the alkaline electrolyte, interlayer anions of LDHs will be rapidly exchanged out by CO_3_^2−^ from CO_2_ in the air, forming regular LDH with CO_3_^2−^, because CO_3_^2−^ shows larger ion‐exchange equilibrium constants and divalent anions are more thermodynamically stable in the LDHs interlayer than monovalent anions.^[^ ^41^ ^]^ It has been reported that replacing the conventional interlayer anions (hydroxide and CO_3_^2−^) of LDHs with other anions could improve the OER performance of LDHs.^[^ ^39b^ ^]^

**Figure 3:**
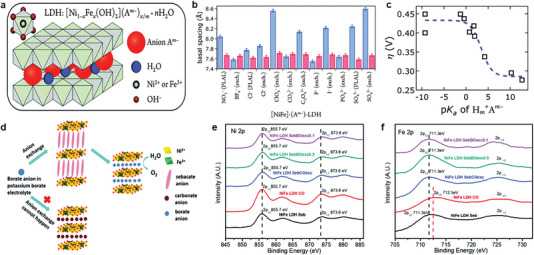
a) Illustration of NiFe‐LDH structure. b) Basal spacings of NiFe‐LDH nanosheets with different interlayer anions as synthesized (blue) and after suspension in 1.0 M aqueous KOH (red). c) Overpotentials η of NiFe‐LDH with different basicity of interlayer anions A^m−^ at 1 mA cm^−2^. Reproduced with permission.^[^ ^23^ ^]^ Copyright 2016, Royal Society of Chemistry. d) Illustration of the in situ anion exchange process for NiFe LDH‐sebacate. e) Ni 2p and f) Fe 2p XPS spectra of the NiFe LDH with different interlayer anions. Reproduced with permission.^[^ ^43a^ ^]^ Copyright 2019, Royal Society of Chemistry.

Anion‐exchange is a viable strategy of anions decoration that not only induces various anions into the interlayer of LDHs, but also facilitate establishing stable intercalation structure to regulate the material properties. To explore the role of interlayer anions in electrochemical water oxidation, Müller and coworkers^[^ ^23^, ^42^ ^]^ took advantage of anion exchange properties of LDHs to prepare NiFe‐LDH with different interlayer anions (NO_3_^−^, BF_4_^−^, Cl^−^, ClO_4_^−^, CO_3_^2−^, C_2_O_4_^2−^, F^−^, I^−^, PO_4_^3−^, or SO_4_^2−^) (**Figure** **3**a,b). The rapid leching of other interlayer anions due to the presence of carbonate has also been reported by others.^[^ ^43^ ^]^ To prevent carbonate in the electrolyte from replacing other interlayer anions, electrolyte was prepared in a virtually CO_2_‐free atmosphere. This research has discovered that local modification of pKa of the interlayer anions strongly influences water oxidation activity of LDH, which shows the sigmoidal dependence on the basicity of interlayer anions (Figure 3c). To overcome structural instability of LDH intercalated with other anions, Komarneni et al^[^ ^43a^ ^]^ synthesized NiFe‐LDH intercalated with sebacate anions and replaced the unstable sebacate anions with borate anions from the pH‐near‐neutral potassium borate electrolyte during OER process (Figure 3d).The in situ borate anions exchange could alter electronic structure of metal to unlock and activate more active sites in the interior layers for superior OER performance (Figure 3e,f).

**Figure 4:**
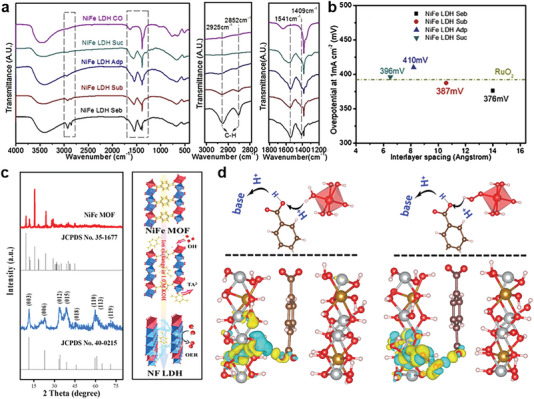
a) FT‐IR spectra of NiFe LDHs intercalated with different dicarboxylic acids. b) Overpotentials of NiFe LDHs with different interlayer spacing in 0.1 M K‐Bi. Reproduced with permission.^[^ ^45^ ^]^ Copyright 2020, Elsevier. c) Left: Powder X‐ray diffraction (PXRD) patterns of original NiFe MOF and sample soaked in alkali solution (blue); Right: schematic diagram of phase transition process of NiFe MOF in alkali solution. d) The differential charge density and illustration of proton transfer processes of NiFe LDH/TA2 for *H_2_O (left) and *OOH (right). Reproduced with permission.^[^ ^46^ ^]^ Copyright 2023, Wiley‐VCH.

The interlayer distance exhibits a close relation to the OER performance of LDHs. In situ anion intercalation can expand the interlayer spacing of LDHs to expose more active sites and accelerate the electrons/ions transportation.^[^ ^44^ ^]^ To systematically investigate the interlayer spacing effects on the electrocatalytic performance, Komarneni et al. fabricated LDHs intercalated with dicarboxylic acids by one‐pot coprecipitation and the interlayer spacing was controlled by employing different dicarboxylic acids.^[^ ^45^ ^]^ FT‐IR spectra reveal that the LDH has been intercalated with dicarboxylic acids of different alkyl chain lengths (**Figure** **4**a). The sebacate anion intercalated NiFe LDH, which had the largest interlayer spacing (14.3 Å), exhibited the highest OER catalytic activities (Figure 4b). Due to the presence of discrepancies in particle sizes and surface areas, the relationship between the interlayer spacing of LDHs and their OER performances requires further exploration.

In the alkaline electrolyte, MOFs is prone to the irreversible hydrolysis of the metal–linker coordination sites, which generates bimetallic LDHs. Meanwhile, during decomposition process, the electron‐rich organic ligands from MOFs remains in the interlayers of LDHs. An impressing work recently reported by Bu's group is to use carboxylic acid‐based MOFs as OER pre‐catalysts to produce NiFe‐LDH that terephthalic acid anion (TA^2−^) are inserted into the second coordination sphere (Figure 4c).^[^ ^46^ ^]^ This work mimics reaction mechanism in photosystem II^[^ ^47^ ^]^ to utilize the synergistic control of active metal center and strong electron‐rich ligands to overcome the inert kinetic bottleneck, greatly enhancing the catalytic activity (Figure 4d).

#### 2.1.3 Different Combination Patterns Between Anionic Groups and Pre‐Catalysts

The combination patterns between anionic groups and pre‐catalysts play a decisive role in surface reconstruction process and the kinetics of electrocatalytic OER processes, which is worthy of further study. The combination patterns includes two general interactions: 1) non‐covalent interactions, including the electrostatic interaction, hydrogen bonding, chemisorption interaction and so on;^[^ ^48^ ^]^ 2) covalent interaction.^[^ ^49^ ^]^ These interactions with anionic ligands effectively modulate the cation active sites, leading to a highly favorable electronic structure that enhances electrocatalytic efficiency.^[^ ^50^ ^]^

##### 2.1.3.1 Electrostatic Interaction

**Figure 5:**
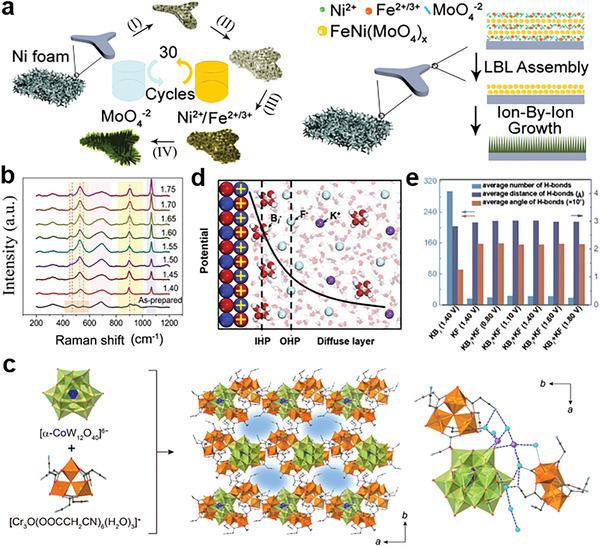
a) Illustration of molybdate oxo‐anionic modification on FeNi surface. b) Operando Raman spectra of FeNi(MoO_4_)_x_@NF. Reproduced with permission.^[^ ^53^ ^]^ Copyright 2022, Wiley‐VCH. c) Left: moleculer structure of the building blocks, middle: crystal structure and right: local structure of of Co‐POM based PIC, blue dashed lines: possible hydrogen bonds. Reproduced with permission.^[^ ^56^ ^]^ Copyright 2022, American Chemical Society. d) Schematic diagram of fluoride (F^−^) and borate (B_i_^−^) anion distribution in electric double layer. e) The results of the hydrogen bonds under different systems and different applied potentials. Reproduced with permission.^[^ ^58^ ^]^ Copyright 2022, Cell Press.

When he surface of pre‐catalysts depicts positive excess free charge density, most anions can be specifically adsorbed since it is electrostatically favorable.^[^ ^51^ ^]^ Under more positive potentials, the adsorption of anions can be enhanced due to electrostatic attraction and the excessive anions coverage may poison catalyst.^[^ ^52^ ^]^ Zhao and co‐workers found that the electrostatic interactions between metallic cations and adsorbed molybdate anions at the multi‐ion layers could provide homogeneous and isotropic nucleation and grain growth during successive ionic layer adsorption and reaction procedure to fabricate amorphous FeNi(MoO_4_)_x_ (**Figure** **5**a).^[^ ^53^ ^]^ During in situ phase transformation process, Raman spectra demonstrated that the partial Mo are dissolved into electrolyte instead of a complete surface reconstruction, indicating molybdate anions remain on the surface (Figure 5b). Additionally, electrostatic effect from anion adsorption have been confirmed to explain the enhanced reaction kinetics.^[^ ^54^ ^]^ And the electrostatic interaction renders its impact on reaction kinetics depending on the type of reaction, oxidation reaction superior to reduction reaction.

##### 2.1.3.2 Hydrogen Bonding

Some specific anions can be anchored on the host catalysts through hydrogen bonding.^[^ ^55^ ^]^ For example, Uchida et al. successfully implement Keggin‐type polyoxometalates (POM) [α‐CoW_12_O_40_]^6−^ and a Cr complex as the building block of porous ionic crystals (PIC) for synergistic catalysis of OER. The polar cyano groups of Cr complex can induce the synergistic influence of Coulomb interactions and hydrogen bonding, which can help highly soluble [α‐CoW_12_O_40_]^6−^ anchored on the crystalline PIC matrix (Figure 5c).^[^ ^56^ ^]^ If the free anions, such as F^−^, Cl^−^, Br^−^ and I^−^ etc., are nearby active sites, the hydrogen bonding of the water‐anion interactions can promote the contact of water with the active centers and thus anions can work as an internal base for proton transfer.^[^ ^57^ ^]^ A representative study by Gong and co‐workers demonstrated that fluoride anions with hydrogen‐bonding characteristics and borate anions with acid‐base reaction characteristics cooperatively enable high water oxidation activity in neutral conditions (Figure 5d).^[^ ^58^ ^]^ Fluoride anions with high affinity can form intermolecular hydrogen bonds with water, which can disturb the original hydrogen‐bonding network and thus create more open space for the borate to further penetrate into the interface to induce more active water molecules (Figure 5e).

##### 2.1.3.3 Chemisorption Interaction

**Figure 6:**
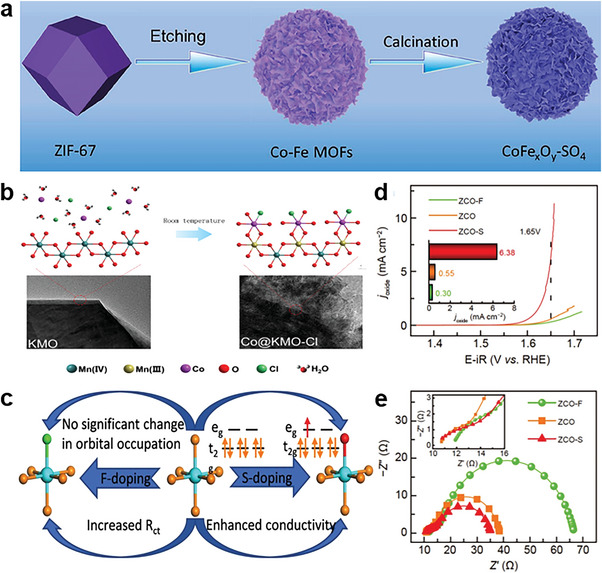
a) Illustration of the CoFe_x_O_y_‐SO_4_ synthesis process. Reproduced with permission.^[^ ^61^ ^]^ Copyright 2023, American Chemical Society. b) Schematic graph of hydrated Co^2+^ reacting with crystalline Mn^4+^. Reproduced with permission.^[^ ^62^ ^]^ Copyright 2021, Elsevier. c) Schematic diagram of anionic modification mechanism. d) LSV curves and e) Nyquist plots of the doped ZCO. Reproduced with permission.^[^ ^69^ ^]^ Copyright 2023, Springer.

Among various combination modes, chemisorption interaction stands out as the most common but intriguing, often referred to as specific anion adsorption. This mode of combination relies on both electronic and chemical forces. The degree of specific anion adsorption on metal surfaces increases with the decreasing energy of solvation.^[^ ^59^ ^]^ The weakly solvated anions, such as SO_4_^2−^, Cl^−^, Br^−^, I^−^ and so forth, can directly construct chemical bonds with the metal surface, contributing to higher ionic surface concentration than that induced by pure electrostatic interaction.^[^ ^60^ ^]^ Recently, Cabot and co‐workers anchored controlled amounts of SO_4_^2−^ anionic groups on the CoFeO_x_ surface with strong chemical bond between anions and metal atoms by exchange of organic ligands (**Figure** **6**a).^[^ ^61^ ^]^ The bidentate bond of SO_4_^2−^ on the surface can increase more acidic sites, which regulates the binding energy of oxygenated intermediates. Rodriguez et al. reported chemical bonds between different anions (chloride, nitrate and sulfate anions) and Co on the layer of CoO_x_H_y_ deposited on the K_0.45_MnO_2_ (Figure 6b).^[^ ^62^ ^]^ As a contrast, strongly solvated species, like F^−^ and ClO_4_^−^, can only be nonspecifically or weakly adsorbed on the host oxide surface. However, mixing anions with different electronegativity can adjust the properties and strength of chemical bonds, which allows the coexistence of chemical bonds (improve conductivity) and covalent bonds (offer structural stability).^[^ ^63^ ^]^ For example, the ionicity of metal‐fluorine bonds can be well adjusted by high covalent metal‐oxygen bonds.^[^ ^64^ ^]^ Due to the strongest electronegativity, the weak metal‐fluorine bonds with the strong ionic nature can be easily formed, which is favorable for surface reconstruction by virtue of structural features of F anions.^[^ ^65^ ^]^

##### 2.1.3.4 Covalent Interaction

Anions with lower electronegativity exhibit greater ease in forming covalent bonds with active metal sites.^[^ ^66^ ^]^ And in the structure of LDH, divalent and trivalent metal cations are combined with the electronegative anions through covalent bonds to assemble electropositive nanoscale laminates.^[^ ^67^ ^]^ The increase of covalent interaction between metal and anions can contribute to the enhancement of preferential attachment of hydroxyl ions on the catalytically active metal sites and thus lowering the overpotential of OER process.^[^ ^66^, ^68^ ^]^ Lu and co‐workers reported that the high covalent interaction of Co‐S trigger the redistribution of electron density from S toward Co, which result in the appearance of Co^2+^ in the sulfur‐doped ZnCo_2_O_4_ (ZCO) (Figure 6c).^[^ ^69^ ^]^ On the contrary, fluorine atoms exhibits strong electronic localization can and strongly drag electrons from metal Co, which causes the weak charge transfer rate in fluorine‐doped ZCO due to the high electronegativity of fluorine (Figure 6d,e). To boost the OER activity of copper‐based catalysts, Yin et al. synthesize Se‐enriched Cu‐Fe‐Se on the copper foam to regulate the covalent bonds in metal‐anion bonds.^[^ ^70^ ^]^

### 2.2 Anions Formed During Electrochemical Process

#### 2.2.1 Anodic Electrooxidation

Anodic electrooxidation irreversibly transforms pre‐catalysts into reconstruction species during anodic oxidation, accompanied by the adsorption of generated anions on the catalyst's surface. According to the sources of anions, anodic electrooxidation can be subdivided into anodization of elements in pre‐catalysts and anodization of elements dissolved in the electrolyte.

##### 2.2.1.1 Anodization of elements in pre‐catalysts

**Figure 7:**
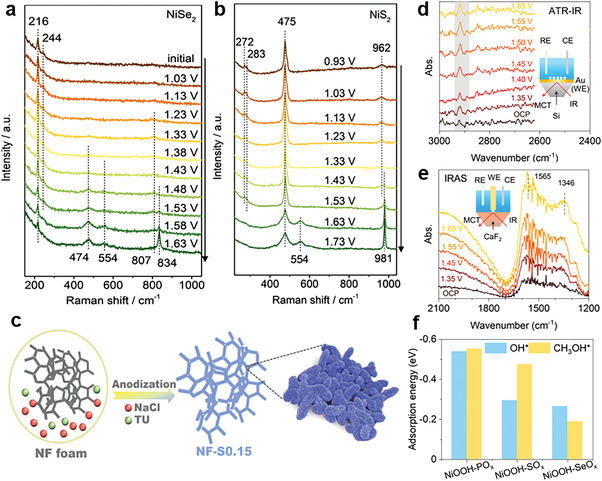
In situ Raman spectra of a) NiSe_2_ and b) NiS_2_ under OER condition. Reproduced with permission.^[^ ^71^ ^]^ Copyright 2020, Wiley‐VCH. c) Schematic diagram of preparing the electrochemical anodized NF‐S0.15. Reproduced with permission.^[^ ^72^ ^]^ Copyright 2021, Wiley‐VCH. d) Operando ATR‐IR spectra and e) IRAS spectra taken on the NiP_x_‐R surface in 0.1 M KOH with 0.5 M methanol. f) Adsorption energies of OH* and CH_3_OH* on NiOOH‐PO_x_, SO_x_ and SeO_x_. Reproduced with permission.^[^ ^74^ ^]^ Copyright 2022, Springer Nature Limited.

Recent research has shown that the oxyanions of SO_4_^2−^, SeO_4_^2−^ and PO_4_^3−^ can be adsorbed on the surface of reconstructed catalysts by in situ anodic electrochemical oxidation of the sulfides, selenides, and phosphides. Anodization of elements in pre‐catalysts has been reported as an innovative strategy to fabricate metal oxyhydroxide modified with anions. Zhang and co‐workers presents NiSe_2_ as the research model to confirm the positive effect of surface‐adsorbed chalcogenate ions from the electrooxidation of transition metal chalcogenides.^[^ ^71^ ^]^ In situ Raman spectroscopy (**Figure** **7**a) monitors the oxidation of Se‐Se to selenite's (SeO_3_^2−^) then to selenates (SeO_4_^2−^). Although the strong signal of selenate is observed in the electrolyte, even a small residual amount of selenate in the activated electrocatalyst plays a crucial role in determining its oxygen evolution reaction (OER) performance. Except for selenates, Zhang et al also investigated if surface‐adsorbed sulfates could exhibit the similar promotion effect(Figure 7b). The Raman spectra and electrochemical results confirm that the promotional effect of surface‐adsorbed chalcogenate ions is general in activated transition metal chalcogenides as OER electrocatalysts.

##### 2.2.1.2 Anodization of elements in electrolyte

The addition in the electrolyte is another vital source for electrochemical anodization to generate oxyanions incorporated into reconstructed species to form the target catalyst. Different from anodization of elements in pre‐catalysts, oxyanions from anodization of elements dissolved in electrolyte are always absorbed on surface of activated catalysts rather than intercalates. As shown in the Figure 7c, Pan et al constructed sulfate ion modified NiFe (oxy)hydroxide via scalable anodization of NiFe foam (NF foam) in the electrolyte with 1.0 M of sodium chloride (NaCl) and 0.15 M of thiourea (TU).^[^ ^72^ ^]^ The adsorbed SO_4_^2−^, which is oxidized S species from TU, can stabilize the intermediate of OOH* on the active site, thus accelerate the OER process.

Additionally, the strategy of anions modulation by in situ anodic electrochemical oxidation of pre‐catalysts is also applicable to other electrochemical oxidation reactions. Li and co‐workers synthesized Se‐Ni(OH)_2_‐shelled vertically oriented NiSe nanowires as a superior electrocatalyst toward urea oxidation reaction through in situ electrochemical oxidation of NiSe nanowires.^[^ ^73^ ^]^ The oxide Se‐Ni(OH)_2_ shell decreases adsorption/desorption barrier of CO_2_ and the rate of reaction is increased. Besides, to optimize methanol electro‐oxidation activity, Wang and co‐workers take advantage of anodic electrochemical oxidation of Ni‐metalloids (NiP_x_, NiS_x_, and NiSe_x_) to tune coordination environment of amorphous nickel oxyhydroxide via different oxyanions, among that the phosphate‐coordinated NiOOH show the highest Ni‐O covalency to promote catalytic activity.^[^ ^74^ ^]^ As shown in the Figure 7d–f, in situ and ex situ characterization results demonstrate that phosphate ions are favorable for the electron transport from the oxygen atoms of OH to Ni species, which could strengthen the adsorption energy of both OH* and CH3OH*, and activate the methanol.

#### 2.2.2 Adsorption of Anion from Electrolyte

**Figure 8:**
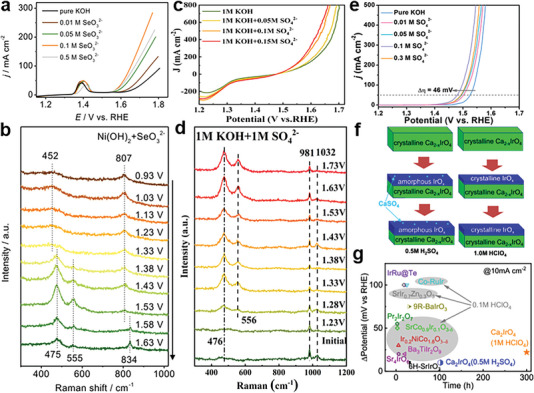
a) LSV of Ni(OH)_2_ in KOH with different concentrations of SeO_3_^2−^. b) In situ Raman spectra of Ni(OH)_2_ in KOH with 0.1 M SeO_3_^2−^. Reproduced with permission.^[^ ^71^ ^]^ Copyright 2020, Wiley‐VCH. c) LSV of Ni_3_S_2_/NF in KOH with different concentrations of SO_4_^2−^. d) In situ Raman spectra of NiOOH in KOH with 1 M SO_4_^2−^. Reproduced with permission.^[^ ^75^ ^]^ Copyright 2022, Elsevier. e) LSV of NF‐S0 in 1 M KOH with different concentrations of SO_4_^2−^. Reproduced with permission.^[^ ^72^ ^]^ Copyright 2021, Wiley‐VCH. f) Scheme of the evolution of the surface structure in 0.5 M H_2_SO_4_ and 1 M HClO_4_, respectively. g) Comparison of the overpotential differentials for pristine Ca_2‐x_IrO_4_ and various state‐of‐the‐art iridium‐based OER oxides. Reproduced with permission.^[^ ^77^ ^]^ Copyright 2022, American Chemical Society.

Adding anions into the electrolytes has also been proven as an efficient and facile strategy for anion modulation on activated catalysts through electrochemical process and then promoting OER kinetics. It is crucial to acknowledge the pH variation of the electrolyte during water oxidation catalysis when introducing anions into the electrolyte. This consideration is necessary to accurately attribute activity enhancements to anion adsorption rather than pH fluctuations. To verify the significant role of the surface‐adsorbed SeO_3_^2−^ in OER performance, Zhang et al. added selenite's into the electrolyte of Ni(OH)_2_ and dramatically improved its OER activity (**Figure** **8**a).^[^ ^71^ ^]^ And the structural transformation of Ni(OH)_2_ is investigated through in situ Raman spectroscopy under 1.0 M KOH with 0.1 M SeO_3_^2−^ (Figure 8b). The works of Pan et al. and Chang et al. both reveal the extra addition of SO_4_^2−^ to KOH solution exhibits a strong driving force for OER activity of activated catalysts.^[^ ^72^, ^75^ ^]^ With the increased concentration of SO_4_^2−^, significant improved activity can be further achieved (Figure 8c–e). However, the excessive coverage of anions possibly blocks part of active sites, degrading the OER performance of anions adsorbed electrocatalysts.^[^ ^76^ ^]^ Thus, an optimal coverage of anions can not only dramatically improve the intrinsic activity of surface‐active sites but also maintain the number of active sites.

The anions adsorption from the electrolyte can induce surface amorphization of electrocatalysts but excessive amorphization can degrade electrochemical long‐term stability as well. Yan and co‐workers reported the comparative studies of our developed Ca_2‐x_IrO_4_ nanocrystals in 0.5 M H_2_SO_4_ and 1 M HClO_4_ electrolytes.^[^ ^77^ ^]^ In the H_2_SO_4_ electrolyte, amorphization layer can be formed on the surface of Ca_2‐x_IrO_4_ nanocrystals after the initial 10 CV cycles due to the binding of Ca^2+^ and SO_4_^2−^ while no obvious surface amorphization can be observed during OER process in the HClO_4_ electrolyte (Figure 8f). The stronger binding strengths of SO_4_^2−^ and CaSO_4_ on Ca_2‐x_IrO_4_ destabilize the nanocrystal surface and promote the surface amorphization, which result in better OER activity and worse stability in H_2_SO_4_ electrolyte than in HClO_4_ electrolyte (Figure 8g).

## 3 Roles of Anion Modulation in Surface Dynamic Reconstruction Process

Compared to other traditional routes of designing novel OER electrocatalysts, anion modulation has recently drawn extensive research interest.^[^ ^78^ ^]^ Current researches are actively committed to confirm the positive effect of anion modulation on the activity and stability of electrocatalysts.^[^ ^79^ ^]^ Thus, to rationally design optimal anion‐incorporated electrocatalysts, the relationship between anion modulation and dynamic reconstruction process should be systematically studied. With recent developments of ex situ and in situ physicochemical characterization instruments and theoretical calculations, it provides the possibility to gain in‐depth insight into the relationship between anion modulation and dynamic reconstruction process.^[^ ^19^ ^]^

### 3.1 Characterization of Anion Modulation During Dynamic Reconstruction

The evolution of pre‐catalysts structure with anion modulation and the formation of new chemical bonds during reconstruction process can be explored at an atomic scale by various spectroscopic characterization techniques, such as Raman spectroscopy,^[^ ^80^ ^]^ FT‐IR spectroscopy,^[^ ^81^ ^]^ mass spectrometry (MS),^[^ ^62^ ^]^ XPS,^[^ ^82^ ^]^ X‐ray diffraction (XRD)^[^ ^83^ ^]^ and X‐ray absorption fine structure spectra (XAFS),^[^ ^84^ ^]^ etc. Furthermore, several typical in situ/operando spectroscopic characterizations can be used to dynamically monitor the oxidation state, geometric and electronic structure, coordination environment, and structural changes of pre‐catalysts with anion modulation during OER process.^[^ ^85^ ^]^

**Figure 9:**
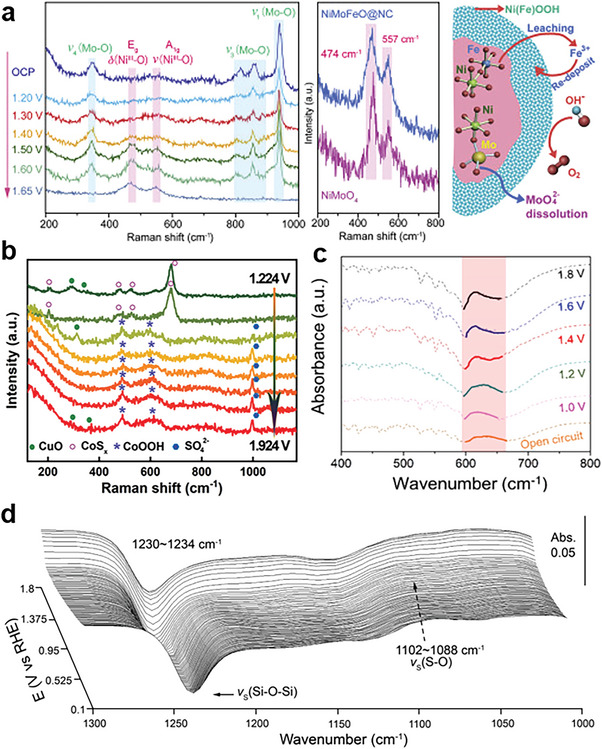
a) Left: in situ Raman spectra of NiMoFeO@NC; Middle: Raman spectra of NiMoFeO@NC and NiMoO_4_ measured at 1.65 V versus RHE; Right: scheme of the self‐reconstruction process for NiMoFeO@NC. Reproduced with permission.^[^ ^88^ ^]^ Copyright 2020, Cell Press. b) In situ Raman spectra of CuO@CoS_x_/CF in 1.0 M KOH solution. Reproduced with permission.^[^ ^89^ ^]^ Copyright 2021, Wiley‐VCH. c) Operando SR‐FTIR spectrum of s‐Ni(OH)_2_. Reproduced with permission.^[^ ^32^ ^]^ Copyright 2021, Elsevier. d) In situ ATR‐SEIRA spectra of sulfate adsorption on Ni anode in 1 M NaOH with 0.5 M NaCl and 0.05 M Na_2_SO_4_. Reproduced with permission.^[^ ^92^ ^]^ Copyright 2021, Wiley‐VCH.

Raman spectroscopy can provide a suite of information about the atomic environment, phase transformation, and the presence of the certain structures through the inelastic scattering of photons due to the vibrations of structures or molecules.^[^ ^86^ ^]^ Surface‐enhanced Raman Spectroscopy (SERS) can strengthen the detection ability for understanding the electrode and electrolyte interface phenomena, where the anion modulation and reconstruction play a critical catalytic role.^[^ ^87^ ^]^ Selomulya et al. employed in situ Raman Spectro‐electrochemistry with ex situ microscopy to detect the dynamic changes of local surface crystal structure of NiMoFeO@NC.^[^ ^88^ ^]^ With the increase of applied bias voltages, the appearance of two spectral features located at 474 and 557 cm^−1^ indicates the formation of γ‐NiOOH and the disappearance of Mo‐O bands at 350, 815–900, and 939 cm^−1^ is ascribed to the leaching of anion MoO_4_^2−^ during reconstruction process (**Figure** **9**a). The in situ Raman spectroscopy demonstrated that the reconstruction process is composed of the leaching of MoO_4_^2−^ and formation of NiOOH together with Fe doping (Figure 9a). Xu's group observed the structure evolution of the pre‐catalysts during the anodic oxidation process via in situ Raman with controlled potentials.^[^ ^89^ ^]^ As the potential is systematically increased, the peaks of new bands corresponding to CoOOH replace that of CoS_x_ accompanying the appearance of the S‐O stretching mode belonging to SO_4_^2−^ (Figure 9b). It indicates that CoS_x_ will be irreversibly oxidized into CoOOH and surface‐adsorbed SO_4_^2−^ during OER process.

FT‐IR spectroscopy can acquire the information about the bonding strength of functional groups and molecular structures based on the direct absorption of photons by bond vibrations.^[^ ^90^ ^]^ Additionally, attenuated total reflection‐surface enhanced infrared absorption spectroscopy (ATR‐SEIRAS) can be used to increase higher signal‐to‐noise ratio and improve the data quality.^[^ ^91^ ^]^ Song and co‐workers performed operando synchrotron radiation FT‐IR (SR‐FTIR) to capture the dynamic anionic behavior of oxidized sulfur species in the s‐Ni(OH)_2_ during self‐reconstruction process.^[^ ^32^ ^]^ An observable absorption band of Ni─S bond ≈600 cm^−1^ along with the increase of potential shows that partial oxide sulfur species can self‐reconstruct into new Ni─S bonds, which reveals that anionic self‐optimization behavior promotes surface reconstruction (Figure 9c). The sulfate adsorption behavior during dynamic reconstruction process was also investigated by Chen's group with the potential dependent in situ ATR‐SEIRAS.^[^ ^92^ ^]^ The absorption band at ≈1102 cm^−1^ was assigned to the symmetric stretching of S = O vibrations from surface‐adsorbed sulfate anion.^[^ ^93^ ^]^ When the potential increased, the phenomenon of red shift happened on the S = O band and meanwhile the S = O band exhibited lower intensity, which reflects the presence of adsorption competition between sulfate anion and oxygenated intermediates during dynamic reconstruction process (Figure 9d).

**Figure 10:**
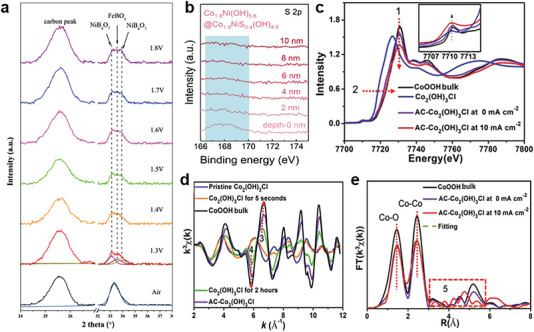
a) In situ SRXRD patterns of NiFeBO_4_ in 1 M KOH. Reproduced with permission.^[^ ^95^ ^]^ Copyright 2021, Springer Nature Limited. b) The depth‐profiling XPS spectra of S 2p. Reproduced with permission.^[^ ^97^ ^]^ Copyright 2019, Wiley‐VCH. c) Normalized Co K‐edge XANES spectra of the Co_2_(OH)_3_Cl and AC‐Co_2_(OH)_3_Cl under realistic OER and non‐OER conditions. d) Co K‐edge XAFS k^3^(χ(k)) oscillation curves of Co_2_(OH)_3_Cl subjected to corresponding activation time. e) Fourier‐transformed Co K‐edge EXAFS spectra for the AC‐Co_2_(OH)_3_Cl. Reproduced with permission.^[^ ^99^ ^]^ Copyright 2019, Wiley‐VCH.

In addition to the spectroscopic techniques mentioned earlier, X‐ray diffraction (XRD) is an accessible and effective method for detecting structural changes resulting from anion modulation and reconstruction.^[^ ^94^ ^]^ Sargent et al. revealed the local crystal structure evolution of NiFe‐boride catalysts by tracking in situ synchrotron radiation XRD (SRXRD) spectra during a chronopotentiometry measurement.^[^ ^95^ ^]^ The changes of spectral features confirmed the phase transformation from the initial structure of NiFeBO_4_ under open circuit potential to the in situ formation of FeBO_3_ and NiB_4_O_7_ under applied positive bias (**Figure** **10**a). As surface‐sensitive spectroscopic characterizations, XPS can measure the surface composition and chemical environments of the elements after anion modulation.^[^ ^96^ ^]^ Based on the depth‐profiling XPS spectra of S 2p, Zhang et al. determined the thickness of amorphous hydroxysulfides prepared by the precisely controllable ethanol‐modified sulfurization strategy.^[^ ^97^ ^]^ The intensity of the S 2p peak weakens along with a deeper probing depth and eventually disappears in bulk phase, which indicates the formation of the hydroxide@hydroxysulfide core‐shell heterostructure through reconstruction process (Figure 10b). XAFS and extended XAFS (EXAFS) offer precise and complementary information on the structural evolution, electronic properties of metal as well as numbers, types, and distances of anion ligands.^[^ ^98^ ^]^ Song's group revealed the electronic and coordination structure changes of Co_2_(OH)_3_Cl during OER process by ex situ and operando XAFS analysis.^[^ ^99^ ^]^ The changes of XAFS and EXAFS spectra reveals the structural disorder of activated Co_2_(OH)_3_Cl (AC‐ Co_2_(OH)_3_Cl) due to the absorption of OER intermediates (Figure 10c,d) and the changed high‐coordination shells in Figure 10e provide direct evidence for the irreversible removal of Cl^−^. The results confirm that the leaching of Cl^−^ is responsible for self‐reconstruction of the catalysts.

### 3.2 Anion Modulation in Manipulating Surface Dynamic Reconstruction Process

**Figure 11:**
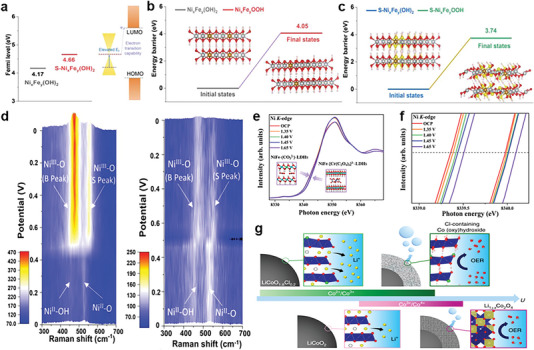
a) Calculated Fermi levels of Ni_x_Fe_y_(OH)_2_ and S‐Ni_x_Fe_y_(OH)_2_. The energy barrier for dehydrogenation of b) Ni_x_Fe_y_(OH)_2_ and c) S‐Ni_x_Fe_y_(OH)_2_ d) Operando Raman spectra of left: S‐NiFe LDH and right: NiFe‐LDH. Reproduced with permission.^[^ ^35b^ ^]^ Copyright 2022, Wiley‐VCH. e) In situ Ni K‐edge XANES spectra of NiFe LDHs and NiFe‐[Cr(C_2_O_4_)_3_]_3_‐LDHs. f) An enlarged view of e). Reproduced with permission.^[^ ^102^ ^]^ Copyright 2022, Elsevier. g) Illustration of surface restructuring process of LiCoO_2_ and LiCoO_1.8_Cl_0.2_ during the OER. Reproduced with permission.^[^ ^20^ ^]^ Copyright 2021, Springer Nature Limited.

High‐valence transition metal oxyhydroxides, formed by in situ electrochemical surface reconstruction, are deemed as the origin of high catalytic activity for OER.^[^ ^21a^ ^]^ Thus, facilitating surface phase transformation to generate metal oxyhydroxide is the prerequisite for efficient OER catalysis. The inner driving force for the reconstruction of pre‐catalysts is the low oxygen vacancy formation energy of activated lattice oxygen, which is correlated with O 2p‐band level relative to the Fermi level.^[^ ^100^ ^]^ Anion substituting oxygen of pre‐catalysts can regulate the O 2p‐band center energy relative to the Fermi level to regulate the degree of surface reconstruction.^[^ ^101^ ^]^ In particular, for LDH materials with ordered electronic configuration, surface reconstruction is limited in only a few nanometers thick layer. Theoretical calculation reveals that S doping gives rise to the higher Fermi level of the S‐Ni_x_Fe_y_(OH)_2_ than that of the Ni_x_Fe_y_(OH)_2_ (**Figure** **11**a). In light of this theoretical prediction, Fan and co‐workers unveiled sulfur partially replacing lattice oxygen to decrease the activation energy barrier of NiFe LDH (Figure 11b,c).^[^ ^35b^ ^]^ Moreover, operando Raman results further demonstrated that S incorporated NiFe LDH nanosheets exhibits a faster phase transformation than pristine NiFe LDH during electrochemical activation (Figure 11d). Furthermore, Wang et al. introduced chromium oxalate into the interlayer of NiFe LDH to create numerous oxygen vacancies. This resulted in the acquisition of more active nickel cations with higher valence states during surface reconstruction. These changes were characterized using in situ XANES (Figure 11e,f).^[^ ^102^ ^]^

Furthermore, anion modulation can redirect the in situ surface reconstruction of pre‐catalysts into a new favorable configuration, which can further enhance OER activity. A representative work by Lim  and co‐workers have reported that Cl doping could alter the structural phase transformation path to yield a favorable formation of oxyhydroxide structure on the surface of cycled Cl‐containing LiCoO_1.8_Cl_0.2_ (Figure 11g).^[^ ^20^ ^]^ Based on the DFT calculations, the anion Cl doped in the layered LiCoO_2‐x_Cl_x_ makes delithiation process be completed under the lower potential, which leads to a larger increase in the Co valence and accelerate stabilization of dynamic reconstruction process.

On the other hand, recent studies have revealed that the rapid etching of anions into electrolyte due to its dissolvable property can give rise to the structural flexibility of pre‐catalysts, facilitating the rapid and deep reconstruction process.^[^ ^30^, ^103^ ^]^ Among various anions, molybdate anion is always applied to decorate the pre‐catalysts to encourage surface reconstruction by dissolution of anions.^[^ ^53^, ^88^ ^]^ For example, Mai et al. prepared the nickel‐based polyoxomolybdate‐organic complex pre‐catalysts and completed the deep construction through the diffusion‐leaching processes of anions.^[^ ^104^ ^]^

## 4 Roles of Survived Anions in Reconstructed Electrocatalysts for the Enhanced Activity

On the ground of experimental characterization and theoretical calculations, many researches have reported all sorts of effect of anion modulation on reconstructed catalysts.^[^ ^22^ ^]^ This section summarizes the different mechanisms of how anion modulation affects reconstructed catalysts and promotes OER performance proposed in the literatures.^[^ ^105^ ^]^ It is worth noting that the following effects are not independent of each other and some of them can work simultaneously.^[^ ^106^ ^]^

### 4.1 Optimizing the Adsorption of Oxygenated Intermediates

**Figure 12:**
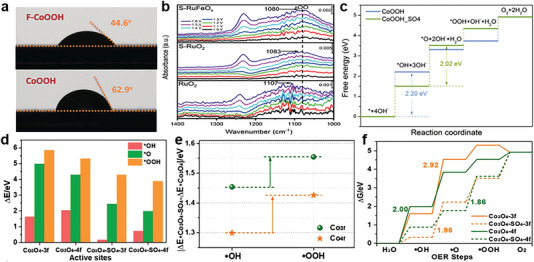
a) Static water contact‐angle results of F‐CoOOH and CoOOH. Reproduced with permission.^[^ ^65^ ^]^ Copyright 2018, Wiley‐VCH. b) In situ ATR‐SEIRAS spectra of S‐RuFeO_x_, S‐RuO_2_, and commercial RuO_2_ in 0.1 M HClO_4_. Reproduced with permission.^[^ ^110^ ^]^ Copyright 2021, Wiley‐VCH. c) Gibbs free energy diagrams of CoOOH and CoOOH‐SO_4_. Reproduced with permission.^[^ ^106^ ^]^ Copyright 2023, Elsevier. d) Adsorption energies of intermediates (*OH, *O, *OOH) on different sites. e) The difference of intermediates adsorption energy between Co_3_O_4_‐SO_4_ and Co_3_O_4_. f) Gibbs free energy diagram of Co_3_O_4_‐SO_4_ and Co_3_O_4_. Reproduced with permission.^[^ ^76^ ^]^ Copyright 2020, Elsevier.

The optimized adsorption energies of oxygenated intermediates (i.e., *OH, *O, and *OOH) play a decisive role in improving the OER kinetics.^[^ ^107^ ^]^ Recently, anion modulation has been regarded as a facile and proven strategy to regulate the adsorption energy of oxygen‐containing intermediates for the improvement of OER performance.^[^ ^108^ ^]^ Additionally, H_2_O molecule adsorption is also widely recognized as a particularly important process.^[^ ^109^ ^]^ Xie et al. conducted contact‐angle measurements to probe that F‐anion‐enriched surface give rise to the higher hydrophilicity of F‐CoOOH for greatly promoting catalytic activity (**Figure** **12**a).^[^ ^65^ ^]^ The experiment or ex situ and in situ/operando characterizations can be employed to assess the adsorption energy of oxygenated intermediates during OER. Zhuang and co‐workers performed in situ ATR‐SEIRAS of RuO_2_, S‐RuO_2_ and S‐RuFeO_x_ to investigate the binding energy of the oxygenated intermediate.^[^ ^110^ ^]^ By the comparison among RuO_2_, S‐RuO_2_ and S‐RuFeO_x_, the redshift of peak, assigned to the stretching vibration of *OO species from the *OO‐H intermediates, reveals the weakened *OO‐H binding of sulfate‐functionalized Ru‐based catalyst (Figure 12b). It is confirmed that the weakening effect, which is in favor of OER process, is ascribed to the surface‐adsorbed sulfate or bisulfate. Besides, the changes in Gibbs free energy (ΔG) for each step of OER pathway, calculated by DFT, can also be used to evaluate, and compare OER activity. Cabot et al. synthesized amorphous ZnCo_x_Ni_y_O_z_ nanosheets decorated with SO_4_^2−^ anion.^[^ ^106^ ^]^ Theoretical calculations indicate that in the presence of sulfate ions, CoOOH exhibits lower adsorption energy of *OOH species, which is the rate‐determining step (Figure 12c).

It is worth noting that the adsorption free energies of oxygenated intermediates possess a strong linear correlation among each other, which constrains the OER activity of active sites.^[^ ^111^ ^]^ Selective stabilization of *OOH over *OH is an important method to break the scaling relation in OER.^[^ ^112^ ^]^ Zou et al. applied solid acid anion formed by sulfate radicals to construct the center of electrophilic acidic sites to break this scaling relationship.^[^ ^76^ ^]^ DFT calculation elucidated that the sulfate anions bonded on active sites enhance adsorption of all these intermediates and result in higher adsorption energy of *OOH than *OH, resulting from hydrogen bonding between SO_4_^2−^ and *OOH, breaks the adsorption‐energy scaling relation (Figure 12d–f).

### 4.2 Tuning Electronic Structure of Active Center

**Figure 13:**
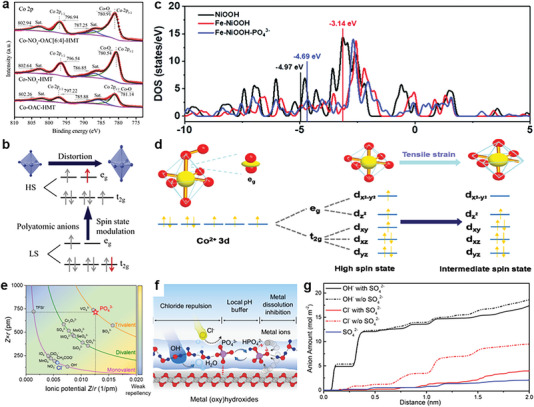
a) XPS Co 2p spectra of Co‐based catalysts. b) The regulation mechanism of polyatomic anions on e_g_ orbital occupancy. Reproduced with permission.^[^ ^120^ ^]^ Copyright 2022, Elsevier. c) DOS curves of the Ni 3d orbitals in NiOOH, Fe‐NiOOH and Fe‐NiOOH‐PO_4_^3−^. Reproduced with permission.^[^ ^121^ ^]^ Copyright 2022, Royal Society of Chemistry. d) Illustration of 3d‐orbital for CoBDC and CoBDC FcCA nanosheets. Reproduced with permission.^[^ ^122^ ^]^ Copyright 2022, American Chemical Society. e) The volcano plot of the repellency between common anions and Cl^−^ ion. f) Illustration of the anti‐corrosion mechanism of surface adsorbed PO_4_^3−^ ions. Reproduced with permission.^[^ ^126^ ^]^ Copyright 2022, Elsevier. g) The amounts of various anions versus the distance above the electrode surface. Reproduced with permission.^[^ ^92^ ^]^ Copyright 2021, Wiley‐VCH.

In recent research, electronic structure of catalysts has been devoted lots of effort to predict the catalytic activity.^[^ ^113^ ^]^ Tuning electronic structure of active center can help to enhance the metallic character,^[^ ^114^ ^]^ increase the hydrophilicity,^[^ ^115^ ^]^ improve the conductivity^[^ ^116^ ^]^ and lower the kinetic barriers^[^ ^117^ ^]^ for the enhanced OER performance.^[^ ^118^ ^]^ The anionic regulation strategy can efficiently regulate electronic structure of active cations due to the strong chemical bond between anions and cations.^[^ ^119^ ^]^ Wang's group prepared the 2D Co‐NO_3_‐OAC[x:y]‐HMT catalyst to modify the electronic structure of active center by the incorporation of NO_3_^−^ and OAC^−^ anions (**Figure** **13**a).^[^ ^120^ ^]^ As shown in Figure 13b, the introduction of NO_3_^−^ and OAC^−^ triggers rearrangement of electrons in cations, inducing a more optimized e_g_ state filling of Co^2+^ with high spin state.

The molecular orbital and energy band structure are both important theories used to correlate electronic structure with OER activity. Recently, Chen et al. modulated the electronic structure and optimized the *d*‐band center of Fe‐NiOOH by PO_4_^3−^ adsorption.^[^ ^121^ ^]^ By contrast, NiOOH with Fe doping and PO_4_^3−^ adsorption exhibits a moderate *d*‐band center (Figure 13c), which decreases the energy barrier of the rate‐determining step (RDS). Interestingly, anionic ligand can also tune the spin state of active metal center. The introduction of ferrocene carboxylic acid (FcCA) into cobalt‐terephthalic acid (CoBDC) catalyst cause a microreactor of tensile strain, which makes high spin state of Co convert to an intermediate spin state (Figure 13d).^[^ ^122^ ^]^ The transformation of spin state has a pivotal role in the O─O bond formation and thereby greatly accelerates the reaction kinetic.

### 4.3 Repulsing Chloride Ions during Seawater Oxidation

One of the most challenging hindrance for seawater oxidation is the existence of chloride anions, which could trigger the competing chloride oxidation reaction and corrode anode catalysts.^[^ ^123^ ^]^ Herein, it is vital to develop the corrosion‐resistive anode catalysts.^[^ ^124^ ^]^ Many studies have supported that anion modulation can increase the energy barrier of the conversion of Cl* to ClO^−^ to protect anode catalysts from the corrosion of chlorine‐containing ions.^[^ ^125^ ^]^ The repellency between chloride ions and other common anions with different charge numbers (Z) and radius (r) has been discussed by Cheng's group to acquire the volcano plot as stated in Figure 13e.^[^ ^126^ ^]^ Comparative study demonstrates that surface‐adsorbed PO_4_^3−^ anions, with optimal ionic potential and the value of Z×r, can construct a soft “semipermeable layer” with H_2_O to inhibit the diffusion of Cl^−^ via coulombic repulsion and alleviate metal dissolution (Figure 13f). In addition, chalcogenide anions have also been confirmed to possess the capacity to regulate the energy barrier of transforming Cl* to ClO^−^.^[^ ^127^ ^]^ For instance, sulfate anion with high electrochemical stability has also been investigated by Chen et al. to retard the corrosion of chloride ions.^[^ ^92^ ^]^ By comparison of system with and without sulfate, the distributions of various anions illustrates that sulfate anion efficiently prevents Cl^−^ from approaching the anode surface without hindering the diffusion of OH^−^ (Figure 13g).

### 4.4 Serving as Proton Acceptor

**Figure 14:**
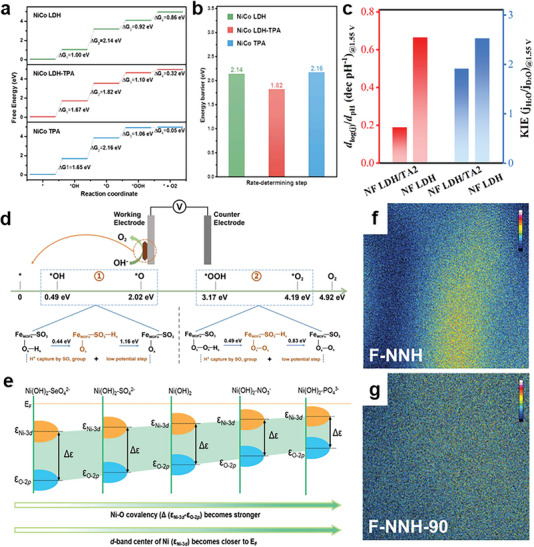
a) Gibbs free energy diagram of NiCo LDH, NiCo LDH‐TPA, and NiCo TPA. b) The energy barriers of the rate‐determining step. Reproduced with permission.^[^ ^133^ ^]^ Copyright 2021, Wiley‐VCH. c) Comparison of pH‐dependent reaction order and KIEs at 1.55 V versus RHE. Reproduced with permission.^[^ ^46^ ^]^ Copyright 2023, Wiley‐VCH. d) Diagram of the thermo‐catalytic process cascaded electrocatalytic pathway over Fe_MOFs_‐SO_3_. Reproduced with permission.^[^ ^134^ ^]^ Copyright 2020, Wiley‐VCH. e) Illustration of oxyanion‐regulated Ni 3d and O 2p band centers for Ni(OH)_2_‐X and pure Ni(OH)_2_ (X = different oxyanions). Reproduced with permission.^[^ ^135^ ^]^ Copyright 2023, Wiley‐VCH. TOF‐SIMS profiles on Fe of f) initial F‐NNH and g) F‐NNH after 90 h CA test at 1.623 V. Reproduced with permission.^[^ ^136^ ^]^ Copyright 2023, Wiley‐VCH.

As multi‐proton‐coupled electron transfer reaction, the kinetics of OER is sluggish because protons are heavier than electrons that leads to the imbalance between electron transfer and proton transfer.^[^ ^128^ ^]^ Therefore, it is crucial to design OER catalysts to increase electrocatalytic efficiency by accelerating O─H bond cleavage.^[^ ^129^ ^]^ Anion modulation strategy can provide electron‐rich ligands as proton acceptor that remove proton from intermediates to balance electron and proton transfer during the process.^[^ ^130^ ^]^ The deprotonation effect of proton acceptor involves two pathways: capturing proton from *OH to form *O^[^ ^131^ ^]^ and weakening the adsorption of the *OO‐H.^[^ ^132^ ^]^ For deprotonation of *OH, Cao et al. induced terephthalic acid (TPA) as principal proton acceptor into NiCo LDH to decrease energy barrier of *O─H bond cleavage.^[^ ^133^ ^]^ From DFT calculations, NiCo LDH‐TPA possesses lower energy barrier for RDS (deprotonation of OH* to O*) than NiCo TPA and NiCo LDH, which manifests the deprotonation ability of TPA anion (**Figure** **14**a,b).

For deprotonation of *OOH, electron‐rich carboxylate anions have been introduced as proton acceptor to mediate proton transfer pathways by Bu's group.^[^ ^46^ ^]^ The pH‐dependence measurements and deuterium kinetic isotope effect experiments (KIEs) provide the support that carboxylate anions, undergoing transition from ‐COO^−^ to ‐COOH, preferentially accepts protons to overcome the inert kinetics of OER (Figure 14c). During thermal catalytic process cascaded electrocatalysis, anionic groups still play a critical role in the deprotonation process. For example, Zhong and co‐workers reported sulfur‐treated Fe‐based MOFs (Fe_MOFs_‐SO_3_) for OER with cascaded thermo‐catalytic processes.^[^ ^134^ ^]^ In consideration of theoretical investigation of Fe_MOFs_‐SO_3,_ protons in the adsorbed *OH and *OOH can both been captured by the ‐SO_3_ group on Fe sites to reduce the overpotential of OER (Figure 14d).

### 4.5 Inhibiting the Segregation of Active Center

Years of studies have presented that the segregation of active center during OER process is one of the most challenging obstacles for transition metal‐based electrocatalysts during the OER process, which would result in the undesirable structural instability. The cause of this obstacle is that oxygenated intermediates could induces leaching of low‐coordinated active metal center into electrolyte.^[^ ^43a^ ^]^ Metal‐oxygen covalency plays a crucial role in catalysts stability in the OER process. The effect of various oxyanions on the OER activity and stability of Ni(OH)_2_ was explored by Wang's group.^[^ ^135^ ^]^ DFT calculations illustrated that different oxyanions incorporation can effectively modulate the energy distance between Ni 3d and O 2p band centers, which is related to the Ni‐O covalency (Figure 14e). By contrast, NO_3_^−^ and PO_4_^3−^ can improve structural stability and enhance OER activity. Ni‐Fe based electrocatalysts suffer from extensive Fe leaching. Suppressing iron segregation is critical to solve the deteriorated stabilities of Ni‐Fe catalysts. Herein, Pan et al. constructed stable FeOOH/Ni_3_(NO_3_)_2_(OH)_4_ with NO_3_^−^ (F‐NNH) in the lattice against Fe dissolution.^[^ ^136^ ^]^ Time of flight secondary ion mass spectrometry (TOF‐SIMS) analysis depicts that NO_3_^−^ ions showcases the inhibition effect on Fe segregation without doubt, which ensures long‐term stability and reduced activity decline (Figure 14f,g).

## 5 Summary and Prospects

Considerable advancement has recommended that the engineering of anion modulation inducing dynamic surface reconstruction represents an emerging strategy to design high‐performance OER electrocatalysts. In this review, we classify the origin of anion modulation into two groups based on the methods of introducing anions and highlights different interaction between anions and pre‐catalysts ranging from covalent bonding to non‐covalent bonding. Furthermore, we present a fundamental understanding of the roles of anion modulation in dynamic reconstruction process and reconstructed electrocatalysts for the enhanced water oxidation activity, as summarized in **Table** **1**. Despite considerable progress achieved by anion modulation, more systematic investigation are required to overcome the current challenges faced:

1. The actual OER active sites should be elucidated more clearly. During the reconstruction process, partial of surface anions are simultaneously oxidized to form other types of anionic groups. Several studies suggest that surface metal compounds tend to self‐reconstruct into M(OH)_x_ or MO_x_H_y_ as real active species and surface anions are completely dissolved in the electrolyte during the electrochemical oxidation.^[^ ^104^ ^]^ On the contrary, other studies deem that the soluble anionic group formed by electrochemical oxidation can re‐adsorb onto the surface of M(OH)_x_ or MO_x_H_y_ and this combination is regarded as actual active sites.^[^ ^71^ ^]^ Furthermore, during reconstruction process, metal‐based compounds can be oxidized into new surface hetero‐anionic structure, which can also work as actual active sites.^[^ ^18^ ^]^ It is thus vitally imperative to determine the bonding strength between anionic groups and reconstruction species as well as the adsorption behavior of reaction intermediates influence by the survived anionic groups. Currently, various in situ/operando characterization techniques can only be employed to investigate the dynamic evolution of surface structure and the adsorption behavior of reaction intermediates on active metal center. Partial anionic groups can participate in the adsorption/activation process of reaction intermediate and collectively promote the catalytic activity for oxygen evolution.
2. It is necessary to develop rational descriptors that can explain how anion modulation further enhances OER activity of reconstruction species. Various descriptors have been established to correlate relationship between the bulk structure and intrinsic activity of OER electrocatalysts. However, anion modulation belongs to surface functionalization for reconstructed electrocatalysts and there is a lack of descriptors that specifically capture the behavior of surface anions. Also, it is worthy to mention that there is an urgent need to consider the reasonable number of anions adsorbed on surface of the active sites for rational design of anion‐modulated electrocatalysts. Excessive adsorption of anionic groups can result in the slower reaction kinetics due to the blocking effect on the active sites. Thus, there is an adsorption competition between the anionic groups and reaction intermediates on the active sites, which has received little attention in current studies.
3. Further research is needed to explore how the different modes of combining anionic groups with reconstruction species influence the OER activity and stability of electrocatalysts in practical applications. When anionic groups are introduced on the surface of electrocatalysts, physical adsorption is more prone to occur than chemical adsorption. However, most studies have focused on investigating chemical adsorption, often overlooking the effects of physical adsorption. Therefore, more efforts should be dedicated to investigating the impact of physical adsorption. Additionally, considering the practical industrial applications of electrocatalysts, it is essential to evaluate the performance of reconstructed catalysts with anion modulation in membrane electrolyzers operating at high current densities. Anion modulation can be achieved by the re‐adsorption of soluble anionic groups from the electrolyte onto the reconstructed species. However, developing electrocatalysts for industrial applications poses significant challenges due to the demanding operating conditions, which involve high temperatures and flowing electrolytes.

**Table 1:** Summary of effects of anion modulation in the different electrocatalyst systems

Pre‐catalysts	Medium	Reconstruction species	Anionic groups	Effect of anion	Ref
NiFe‐based SURMOFs	0.1 M KOH	NiFe (oxy)hydroxide	terephthalic acid	Tune electronic structure of active center	[24]
NiSe_2_	1 M KOH	Ni(OH)_2_	SeO_4_^2−^	Optimize the adsorption of oxygenated intermediates	[71]
NiFe foam	1 M KOH	NiFe (oxy)hydroxide	SO_4_^2−^	Accelerate the electrochemical reconstruction; Optimize the adsorption of oxygenated intermediates	[72]
Ni‐metalloids	1 M KOH	NiOOH	TO_x_: T = P, S, and Se	Optimize the adsorption of oxygenated intermediates; Tune electronic structure of active center	[74]
Co_3_O_4_	1 M KOH	Co (oxy)hydroxide	SO_4_^2−^	Optimize the adsorption of oxygenated intermediates; Tune electronic structure of active center	[76]
NiMo foam	1 M KOH	Fe incorporated NiMo oxyhydroxide	MoO_4_^2−^	Accelerate the electrochemical reconstruction; Optimize the adsorption of oxygenated intermediates	[28]
NiMoO_4_	1 M KOH	NiOOH	MoO_4_^2−^	Accelerate the electrochemical reconstruction	[30b]
Ni_1.5_Sn@triMPO_4_	1 M KOH	Ni(Fe)OOH	PO_4_^3−^	Optimize the adsorption of oxygenated intermediates	[33]
S‐Ni_x_Fe_y_(OH)_2_	1 M KOH	Ni_x_Fe_y_OOH	S	Optimize the adsorption of oxygenated intermediates	[35b]
NiFe LDH	0.1 M potassium borate	NiFe (oxy)hydroxide	sebacate anions	Tune electronic structure of active center	[43a]
NiFe LDH	0.1 M potassium borate	NiFe (oxy)hydroxide	dicarboxylate anions	Tune electronic structure of active center	[45]
Carboxylic acid‐based MOFs	1 M KOH	NiFe LDH	carboxylate anions	Serve as proton acceptor	[46]
Ni_3_S_2_/NF	1 M KOH	NiOOH	SO_4_^2−^	Optimize the adsorption of oxygenated intermediates	[75]
FeNi(MoO_4_)_x_@NF	1 M KOH	γ‐FeOOH/β‐NiOOH	MoO_4_^2−^	Optimize the adsorption of oxygenated intermediates	[53]
CoF_2_	1 M KOH	CoOOH	F	Optimize the adsorption of oxygenated intermediates	[65]
NiMoFeO@NC	1 M KOH	NiFeOOH	MoO_4_^2−^	Accelerate the electrochemical reconstruction	[88]
Nickel foam	1 M NaOH + 2.5 M NaCl	NiOOH	SO_4_^2−^	Repulse chloride ions during seawater oxidation	[92]
NiFe‐Boride compound	1 M KOH	NiB_4_O_7_	B_4_O_7_^2−^	Optimize the adsorption of oxygenated intermediates	[95]
Co_2_(OH)_3_Cl	1 M KOH	Co (oxy)hydroxide	Cl^−^	Accelerate the electrochemical reconstruction	[99]
NiFe‐POMo nanowires	1 M KOH	NiFe (oxy)hydroxide	[Mo_8_O_26_]^4−^	Accelerate the electrochemical reconstruction	[104]
NiFe LDH	1 M KOH	NiFe (oxy)hydroxide	[Cr(C_2_O_4_)_3_]^3−^	Tune electronic structure of active center	[102]
LiCoO_2‐x_Cl_x_	1 M KOH	Co (oxy)hydroxide	Cl	Redirecting dynamic surface reconstruction	[20]
Co‐NO_3_‐OAC [x:y]‐HMT	1 M KOH	Co (oxy)hydroxide	CH3COO^−^/ NO_3_^−^	Tune electronic structure of active center	[120]
Fe, P–NiCH/CNTs	1 M KOH	NiOOH	PO_4_^3−^	Tune electronic structure of active center	[121]
Cobalt‐terephthalic acid	1 M KOH	Co (oxy)hydroxide	ferrocene carboxylic acid	Tune electronic structure of active center	[122]
NiFe LDH	1 M KOH + 0.5 M NaCl	NiFe (oxy)hydroxide	PO_4_^3−^	Repulse chloride ions during seawater oxidation	[126]
NiCo LDH	1 M KOH	NiCo (oxy)hydroxide	terephthalic acid	Serve as proton acceptor	[133]
Ni foam	1 M KOH	Ni(OH)_2_	NO_3_^−^, PO_4_^3−^, SO_4_^2−^, SeO_4_^2−^	Inhibiting the segregation of active center	[135]
Ni_3_(NO3)_2_(OH)_4_	1 M KOH	FeOOH	NO_3_^−^	Inhibiting the segregation of active center	[136]

Despite the challenges that lie ahead in the development of anion modulation inducing dynamic surface reconstruction, significant progress can be expected by synergistically combining advanced operando characterization techniques and molecular dynamics simulations in the future. This approach will enable the clear and quantitative analysis of the surface evolution of electrocatalysts, bridging the gap between experimental observation and practical application. In summary, a comprehensive and in‐depth understanding of anion modulation inducing dynamic surface reconstruction holds great potential for achieving remarkable advancements in the field of water oxidation and other electrocatalytic reactions.

## Conflict of Interest

The authors declare no conflict of interest.
